# Multi-differentiation potential is necessary for optimal tenogenesis of tendon stem cells

**DOI:** 10.1186/s13287-020-01640-8

**Published:** 2020-04-09

**Authors:** Ibtesam Rajpar, Jennifer G. Barrett

**Affiliations:** Department of Large Animal Clinical Sciences, Marion duPont Scott Equine Medical Center, Virginia-Maryland College of Veterinary Medicine, Virginia Tech, Leesburg, 17690 Old Waterford Road, Leesburg, VA 20176 USA

**Keywords:** Mesenchymal stem cell, Tendon, Tendon stem/progenitor cells, Collagen gel, Differentiation, Tissue engineering

## Abstract

**Background:**

Tendon injury is a significant clinical problem due to poor healing and a high reinjury rate; successful treatment is limited by our poor understanding of endogenous tendon stem cells. Recent evidence suggests that adult stem cells are phenotypically diverse, even when comparing stem cells isolated from the same tissue from the same individual, and may in fact exist on a spectrum of proliferation and differentiation capacities. Additionally, the relationships between and clinical relevance of this phenotypic variation are poorly understood. In particular, tenogenic capacity has not been studied in comparison to tenogenic differentiation and cell proliferation. Toward this end, we performed a comprehensive assessment of cell proliferation and differentiation capacity toward four connective tissue lineages (tendon, cartilage, bone, and adipose) using tendon stem cell lines derived from single cells released directly from tendon tissue to (1) evaluate the differences, if any, in tenogenic potential, and (2) identify the relationships between differentiation phenotypes and proliferation capacity.

**Methods:**

Tendon stem cells were derived from the endotenon of superficial digital flexor tendon from 3 horses. The cell suspension from each horse was separately plated simultaneously (1) at moderate density to generate a heterogenous population of cells—parent tendon cell line—and (2) at low density to separate single cells from each other to allow isolation of colonies that derive from single mother cells—clonal tendon stem cell lines.

Thirty clonal tendon stem cell lines—10 from each horse—and each parent tendon cell line were assessed for tenogenesis, tri-lineage differentiation, and cell proliferation. Differentiation was confirmed by lineage-specific cell staining and quantified by the relative gene expression of lineage-specific markers. Statistical significance was determined using analysis of variance and post hoc Tukey’s tests.

**Results:**

Three distinct differentiation phenotypes—differentiation potency toward all 4 tissue lineages and two tri-lineage differentiation potencies—were identified in tendon clonal stem cell lines. These phenotypes were differentiation toward (1) tendon, cartilage, bone, and adipose (TCOA); (2) tendon, cartilage, and bone (TCO); and (3) tendon, cartilage, and adipose (TCA). Further, clonal cell lines that differentiated toward all four lineages had the highest expression of scleraxis and mohawk upon tenogenesis. Moreover, cell proliferation was significantly different between phenotypic groups, as evidenced by increased numbers of cumulative cell population doublings in clonal cell lines that did not differentiate toward adipose.

**Conclusions:**

Our study provides evidence of the heterogenous character of adult stem cells and identifies key differences in tendon stem cell differentiation and proliferative potentials from the same individual and from the same tendon. Isolation of tendon stem cell lines with the capacity to differentiate into all four connective tissue lineages may yield improved therapeutic benefits in clinical models of repair and promote a native, regenerative phenotype in engineered tendons. Future studies may be targeted to understanding the functional contributions of each tendon stem cell phenotype in vivo and identifying additional cell phenotypes.

## Introduction

Tendon injuries are debilitating and significantly impact quality of life. Over 32 million cases of musculoskeletal injuries involving tendon are reported every year in the USA, and the incidence of tendon injuries is increasing [1]. Acute and chronic injuries of the Achilles, patellar, and rotator cuff tendons are most prevalent in the adult population. Acute injuries are common in athletes, whereas chronic injuries usually arise from tendon overuse or aging [2, 3]. Tendons heal poorly [4], and surgical intervention is often necessary to augment healing. However, the incidence of re-injury following surgical repair can be as high as 20–60% [5]. More recently, regenerative therapies using platelet-rich plasma or mesenchymal stem cells (MSCs) have shown promise in the restoration of native tendon structural and mechanical properties [6]. Among these, bone marrow-derived MSCs are generally preferred; they are easily accessible, well characterized, and have been efficacious in in vivo models [7]. However, bone marrow-derived MSCs have also formed bone after implantation, may not optimally differentiate into tenocytes, and may require pre-transplantation conditioning in bioreactors or with growth factors to facilitate tendon regeneration. Thus, an alternate stem cell source such as tendon may be better suited for regenerative tendon healing [8].

Endogenous tendon stem/progenitor cells (TSCs) arise from the tendon progenitor niche that is predominantly a collagen I-rich extracellular matrix [9]. TSCs are the principal mediators of key processes involved in tendon repair, such as control of the inflammatory response and the synthesis and remodeling of collagen [10, 11]. Like bone marrow-derived MSCs, TSCs express stem cell markers, have high proliferative capacity, and can differentiate to non-tendon lineages in vitro [12]. Recent studies suggest that TSCs from anatomically different regions of tendon such as the peritenon and the tendon core (endotenon) exhibit differences in their morphologies and tendon healing potentials [11, 13]. For example, TSCs from endotenon express higher message levels of scleraxis and tenomodulin than those from the peritenon, whereas the reverse was shown for the pericyte marker CD133 [8]. This suggests that more than one type of TSC may reside in tendon tissue. Even within the same stem cell type, phenotypic variation has been documented, based on individual variation between donors, variation between clonal stem cells from the same individual, as well as age of cells at the time of collection [9, 14–16]. A comprehensive analysis of TSC lines generated from individual TSC cells isolated directly from endotenon is required to understand the molecular basis of these differences and their contribution to tendon healing in vivo*.*

Phenotypic variation of TSCs challenges the current paradigm of one phenotype that encompasses all MSCs; thus, the criteria proposed by Dominici et al. may not define TSCs [17, 18]. The study of clonal TSC lines has improved our understanding of TSC heterogeneity. Bi et al. reported tri-, bi-, and uni-potential TSCs, based on their results from tri-lineage differentiation of human clonal TSC lines [9]. However, this study did not evaluate tenogenic capacity of TSCs, and benchtop assays of tri-lineage differentiation may be poor indicators of tenogenic potential. Thus, it is unknown whether individual TSCs that are tri-, bi-, and uni-potential in the tri-lineage assays differ in their tenogenic potential.

Our goals in this study were to determine cell proliferation capacity and differentiation potential of clonal TSC lines toward four lineages—tendon, cartilage, bone, and adipose—and to identify TSC composite differentiation phenotypes (uni-, bi-, tri-, and/or quadri-potential cell lines) based on these differentiation assays and relate them to proliferative capacity. In addition to standard population doublings and tri-lineage differentiation assays, we incorporated a previously reported tenogenesis assay to assess TSC ability to form their tissue of origin [19]. The tenogenesis assay comprises cells suspended in type I collagen hydrogel with tenogenic growth factors and static strain provided by cell-gel contraction around static cylinders. Differentiation was confirmed by lineage-specific cell staining and quantified by gene expression analysis of lineage-specific markers. Tenogenesis was further quantified by gel contraction and histomorphometry. We hypothesized that clonal cell lines of TSCs would demonstrate heterogenous cell proliferation and differentiation phenotypes from one another. We further hypothesized that TSCs that differentiate into four connective tissue lineages would have optimal tenogenic capacity.

## Materials and methods

### Experimental design

**Fig. 1:**
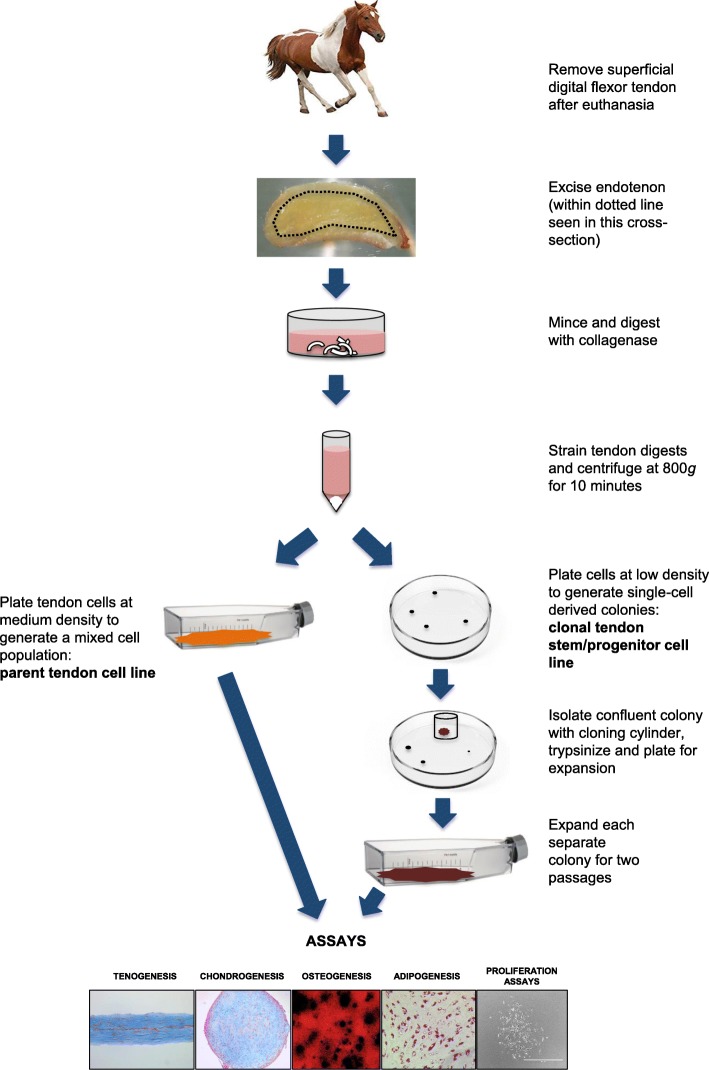
Cell culture workflow

TSCs from three juvenile horses (*N* = 3) were included in this study. Each parent tendon cell line was analyzed for the gene expression of the stem cell markers CD90, CD105, GNL3, and Oct-4 on day 0 and at confluence. Thirty clonal TSC lines were generated by plating cells at low density on culture vessels and expanding in culture for two passages (Fig. 1) [20]. Clonal lines were analyzed for gene expression of the transcription factor Oct-4 in monolayer cultures on day 21. Cell population doubling assays, tri-lineage differentiation assays, and a tenogenesis assay were performed on clonal TSC lines. Positive differentiation was determined based on staining assays for each tissue type, as well as upregulation of gene expression (gene amplification) of scleraxis, mohawk, decorin, biglycan, collagen III, tenascin C, Axin2, and fibroblast-specific protein-1 (FSP1) for tenogenesis, Sox9, and aggrecan for chondrogenesis, Runx2 for osteogenesis, and fatty acid binding protein-4 (FABP4) for adipogenesis after cells were grown under differentiation conditions.

Clonal TSC lines were categorized based on their differentiation potentials, which were determined from the results of each lineage-specific differentiation assay. Hence, each clonal TSC line was designated with a letter for each positive differentiation outcome, specifically T = tenogenesis positive, C = chondrogenesis positive, O = osteogenesis positive, and A = adipogenesis positive. Each cell line was grouped with other cell lines exhibiting the same differentiation potential. Between-group comparisons were performed for statistical analysis of gene expression, gel contraction, cell alignment, and population doublings.

### Cell isolation and clonal cell line preparation

A complete cell culture workflow is illustrated in Fig. 1. Tendon samples from each horse were processed independently to generate three independent parent tendon cell lines and thirty clonal tendon stem cell lines (10 per horse). Immediately after euthanasia and with IACUC approval, thoracic limb superficial digital flexor tendons (*N* = 3) were excised from horses aged 1 month (horse 112111), 3 months (horse 111961), and 12 months (horse 111755). Endotenon was dissected away from overlying paratenon and epitenon (Fig. 1). Endotenon from each tendon was separately minced and digested with collagenase overnight at 37 °C. The next day, endotenon debris was removed from each suspension using a 100-μm filter and cells were pelleted via centrifugation at 800*g* for 10 min. Cells were resuspended in growth media and plated directly into separate tissue culture flasks at (1) medium density of 6666 cells/cm^2^ to derive heterogenous parent TC lines and (2) low density of 111 cells/cm^2^ to obtain single-cell-derived clonal colonies. All cell lines were cultured in growth media comprising high-glucose DMEM (Thermo Scientific), 10% Cellect™ Silver fetal bovine serum (MP Biomedicals, Santa Ana, CA), and 1% penicillin-streptomycin solution (Thermo Scientific). Single cells were monitored for formation of colonies, and those colonies were isolated from each other using 10 mm cloning cylinders (0.8 × 0.8 cm, Corning, Inc., Corning, NY) [20]. Each colony was detached separately using trypsin-EDTA [21]. For the experiment, clonal cell lines were expanded in culture for two successive passages before plating for differentiation assays and population doublings. Only cell lines that grew colonies that became confluent in the cloning cylinders were included in the study, due to the requirement of numerous cells for the assays.

### Cell proliferation

Ten thousand cells per cell line were plated in individual wells of 24-well tissue culture plates (Nunc™, Thermo Scientific), trypsinized at 70–80% confluence, and counted with an automated cell counter (Beckman Coulter, Brea, CA). Population doubling numbers (DN) and doubling time (DT) for each passage and cumulative doubling numbers (CDN) over 3 successive passages were calculated using the following formulae:

$$ \mathrm{DN}={\log}_2\left(\mathrm{cell}\ \mathrm{number}\ \mathrm{at}\ \mathrm{confluence}/\mathrm{cell}\ \mathrm{number}\ \mathrm{at}\ \mathrm{seed}\right) $$

$$ \mathrm{DT}\ \left(\mathrm{days}\right)=\mathrm{culture}\ \mathrm{duration}\times \log (2)/\log\ \left(\mathrm{cell}\ \mathrm{number}\ \mathrm{at}\ \mathrm{confluence}/\mathrm{cell}\ \mathrm{number}\ \mathrm{at}\ \mathrm{seed}\right) $$

$$ \mathrm{CDN}=\mathrm{sum}\ \mathrm{of}\ \mathrm{DNs}\ \mathrm{from}\ \mathrm{each}\ \mathrm{passage} $$

### Tenogenesis assay

Gels for tenogenesis were generated using a previously published method [19] and grown in tenogenic growth media comprising high-glucose DMEM (Thermo Scientific), 10% Cellect™ Silver fetal bovine serum (MP Biomedicals), 37.5 μg/ml l-ascorbic acid (Sigma-Aldrich, St. Louis, MI), and 1% penicillin G (Sigma-Aldrich). Briefly, one million cells were suspended in 5 ml of an immediately mixed solution of rat tail collagen I (10 mg/ml solution from Corning Life Sciences, Tewksbury, MA) diluted to a final concentration of 0.8 mg/ml in tenogenic growth media with suspended cells buffered to pH = 7.0. Cell/gel suspensions were plated immediately into individual wells of 4-well rectangular dishes (Nunc™, 12.8 × 8.6 cm, Thermo Scientific) with two sterile cloning cylinders (0.8 × 0.8 cm, Corning Inc.) fixed in place 3 cm apart from each other along the longitudinal midline of the well. This configuration results in tenogenic cells contracting the gel and forming a tendon-like construct. Gels were maintained at 37 °C, with 5% CO2 and 90% humidity. On day 1, growth media was replaced with media containing 50 ng/ml BMP-12 (recombinant human, Sigma Aldrich) and 10 ng/ml IGF-1 (recombinant human, BioVision, San Francisco, CA). Media was changed on alternate days over a 10-day period.

### Gel histology and analysis of cell alignment

Longitudinal sections of each gel were fixed in 4% paraformaldehyde overnight at 4 °C, washed in phosphate-buffered saline the next day, and submitted for histology to a commercial service (Laudier Histology, New York, NY). Two 6-μm-thick longitudinal slices per sample were stained with Masson’s trichrome stain, and images were acquired with a microscope (Olympus Corp, Center Valley, PA) and digital camera (Motic North America, Richmond, BC). Cell alignment was quantified using ImageJ software analytical tools [22]. Fifty cellular angles per histological section and two sections per sample were measured relative to the longitudinal gel axis. Parallel alignment to the longitudinal axis was assigned 0°, and angles of each cell relative to 0° (0–90°) were averaged for each sample to draw comparisons between samples.

### Gel contraction

Digital photographic images of each gel were taken on days 1, 3, 5, 7, and 10 to determine the percentage of contracted area at each time point relative to the gel area at day 0. Images were analyzed using ImageJ software analytical tools.

### Tri-lineage differentiation

Tri-lineage differentiation potential was assessed using standard benchtop assays of adipogenesis, osteogenesis, and chondrogenesis using protocols and time points that were optimized for equine MSCs prior to conducting this study to limit the total number of clonal tendon stem cells needed for each assay. Equine bone marrow-derived MSCs and equine tendon stem cells were each used to assess optimum time point (compared days 1, 7, 14, and 21) and genes for analysis of differentiation. We compared the following: (1) for proteoglycan staining of cartilage pellets and gene expression of aggrecan, collagen type II and Sox9; (2) for osteogenesis Alizarin Red staining of cell matrix and gene expression of osteocalcin, osteopontin and Runx2; and (3) for adipogenesis, Oil Red O staining of cells and gene expression of PPARγ, FABP2, and FABP4. We had determined that on day 21, cell and matrix staining was optimal for all three, with the following genes most highly expressed: Sox9 and aggrecan for chondrogenesis, Runx2 for osteogenesis, and FABP4 for adipogenesis; therefore, we used the 21-day time point and expression of those genes for the tri-lineage differentiation assays.

For adipogenesis and osteogenesis, cells were plated at high (21,000 cells/cm^2^) and low densities (4000 cells/cm^2^) respectively in tissue culture dishes (Nunc™, Thermo Scientific) and cultured in growth media comprising high-glucose DMEM and 10% fetal bovine serum (Thermo Scientific). At 70–80% culture confluence, media was replaced with differentiation media and maintained for 21 days.

For chondrogenesis, 200,000 cells per cell line were centrifuged at 800*g* for 10 min to obtain a pellet. Pellet cultures were maintained in growth media comprising high-glucose DMEM and 1% insulin-transferrin-selenium mix (Gibco™, Thermo Scientific) for 2 days prior to differentiation for 21 days.

Differentiation media comprised the following: for adipogenesis, StemPro™ adipogenesis differentiation medium (Thermo Scientific); for osteogenesis, growth media supplemented with 10 mM beta-glycerophosphate, 50 μg/ml ascorbate 2-phosphate, and 100 nM dexamethasone (all Sigma-Aldrich); and for chondrogenesis, growth media supplemented with 37.5 μg/ml ascorbate 2-phosphate, 100 nM dexamethasone (both Sigma Aldrich), and 10 ng/ml TGF-β3 (recombinant human, R&D Systems, Minneapolis, MA). On day 21, cultures were either fixed with 10% formalin (Sigma Aldrich) or frozen for gene expression analysis. Positive results from gene expression analysis were confirmed with Oil Red O (for adipogenesis), Alizarin Red S (for osteogenesis) staining (both Sigma Aldrich), and Alcian Blue staining of chondrocyte pellets, and images were acquired using an inverted microscope (Olympus Corp, Center Valley, PA).

### Gene expression

RNA isolation was performed using the TRIzol™ method (Thermo Scientific). RNA pellets were subjected to RNeasy® spin columns for removal of genomic DNA contamination (QIAGEN Inc., Germantown, MD), and purified RNA was quantified using a NanoDrop™ 2000c spectrophotometer. cDNA was synthesized using a commercial kit (High-Capacity RNA-to-cDNA kit, Thermo Scientific). Real-time qPCR (7500 Real-Time PCR System, Thermo Scientific) was performed using custom TaqMan®-MGB probes and primers (Thermo Scientific) included in Table 1. TaqMan® gene expression assays for equine-specific Runx2 (Assay ID: Ec03469741_m1), Sox9 (Assay ID: Ec03469763_s1), Axin2 (Assay ID: APT2CHG), and FSP-1 (Assay ID: APU643E) were obtained from Thermo Scientific. The comparative threshold cycle method (2^−ΔΔCt^) was employed for the relative quantification of gene expression [23]. Data were normalized to GAPDH. Tenogenic marker expression is reported as fold change with respect to an equine juvenile tendon reference control. Tri-lineage marker expression is reported as fold change relative to monolayer controls cultured in growth media on day 21. Stem cell marker expression is reported as fold change relative to an adult equine muscle negative control for parent TC lines and relative to day 0 parent TC lines (monolayer culture) for clonal TSC lines.

**Table 1:** Custom-designed equine primer and probe sequences or Assay ID numbers for pre-designed sets

	Forward	Reverse	Probe
**GAPDH**	CAAGTTCCATGGCACAGTCAAG	GGCCTTTCCGTTGATGACAA	CCGAGCACGGGAAG
**GNL3**	TTCGGGAAGCTGAGCTAAGG	CTGTCAAGCTTCTGCTGCTGTT	AACAGCGGCTTGAAG
**CD90**	GGCAGACCAGAGCCTTCGT	ATGGGTGTGGCGGTGGTAT	TGGACTGCCGCCATG
**CD105**	TCCACATCCTCTTCCTGGAGTT	GGACCTTTGGATAGTCAGCTTCA	CCAAGGGATGTGTCAGAG
**Oct-4**	CAGCTCGGGCTCGAGAAG	TTCTGGCGACGGTTGCA	ACGTGGTACGAGTGTGG
**FABP4**	AAAATCCCAGAAACCTCACAAAAT	TCACTGGCGACAAGTTTCCA	TGTGATGCATTTGTAGGCA
**Runx2**	Assay ID: Ec03469741_m1		
**Sox9**	Assay ID: Ec03469763_s1		
**Aggrecan**	GACCACTTTACTCTTGGCGTTTG	GTCAGGGTCTGAAACGTCTACTGA	ACTCTGAGGGTCATCAC
**Scleraxis**	CGCCCAGCCCAAACAG	TTGCTCAACTTTCTCTGGTTGCT	TCTGCACCTTCTGCC
**Mohawk**	CCCACCAAGACGGAGAAGATACT	CACCTGCACTAGCGTCATCTG	TTGGCGCTCGGCTC
**Collagen III**	CTGCTTCATCCCACTCTTAT	ATCCGCATAGGACTGACCA	AACAGGAAGTTGCTGAAGG
**Collagen I**	GCCAAGAAGAAGGCCAAGAA	TGAGGCCGTCCTGTATGC	ACATCCCAGCAGTCACCT
**Decorin**	AAGTTGATGCAGCTAGCCTG	GGCCAGAGAGCCATTGTCA	ATTTGGCTAAATTGGGACTG
**Biglycan**	TGGACCTGCAGAACAATGAGAT	AGAGATGCTGGAGGCCTTTG	TCTGAGCTCCGAAAGG
**Tenascin C**	GTTGGACTCCTGTACCCATTCC	GGCCCGAGGTCGTGTCT	TCCCAAGCGATGCTG
**FSP-1**	Assay ID: APU643E		
**Axin2**	Assay ID: APT2CHG		

### Statistical analysis

Non-normal data were log transformed prior to analysis. Clonal TSC line differentiation potential was classified into differentiation capacity phenotypic groups, as described in the “Experimental design” section. Group means were compared to each other using a one-way ANOVA and post hoc Tukey’s tests for gene expression, population doublings (number and time), and cell alignment. Significant differences in contraction were assessed using a one-way MANOVA with a repeated measures design and post hoc Tukey’s tests. A *p* value of less than 0.05 was considered significant. Computation was performed in JMP Pro 15 (SAS Institute, Cary, NC) and MS Excel 11 (Microsoft, Redmond, WA).

## Results

### Clonal TSC lines proliferate in two- and three-dimensional culture

**Fig. 2:**
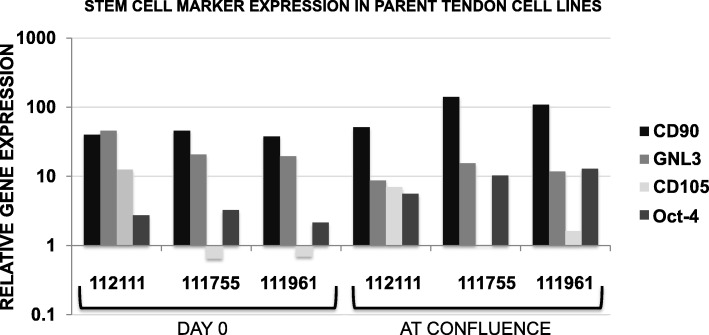
Gene expression of stem cell markers in parent tendon cell lines: 112111, 111755, and 111961 are individual subject identifiers. CD90 and CD105 are cell surface proteins, GNL3 is a nuclear protein, and Oct-4 is a transcription factor

All parent tendon cell lines used in this study expressed the stem cell markers CD90, CD105, GNL3, and Oct-4 in monolayer culture at day 0 and at confluence (Fig. 2). Of the thirty clonal colonies isolated and expanded in culture, twenty-six yielded a million cells or more at confluence and were seeded in 3D hydrogels for analysis of tenogenic potential. Of these, fifteen clonal TSC lines were additionally seeded for tri-differentiation assays and cultured in growth media to assess population doublings. Thirteen out of fifteen clonal TSC lines expressed Oct-4 in monolayer culture on day 21 (Additional Fig. 1). TSCs successfully adhered to tissue culture-treated plastic, formed 3D pellets for chondrogenesis, and expanded to confluence in successive monolayer and 3D cultures. Data from fifteen clonal TSC lines were analyzed for comparisons of differentiation and proliferative potentials, since enough cells could be procured from a single passage of each of these cell lines to enable four differentiation assays and one assay of population doublings.

### Three distinct TSC phenotypes can be found in tendon

**Fig. 3:**
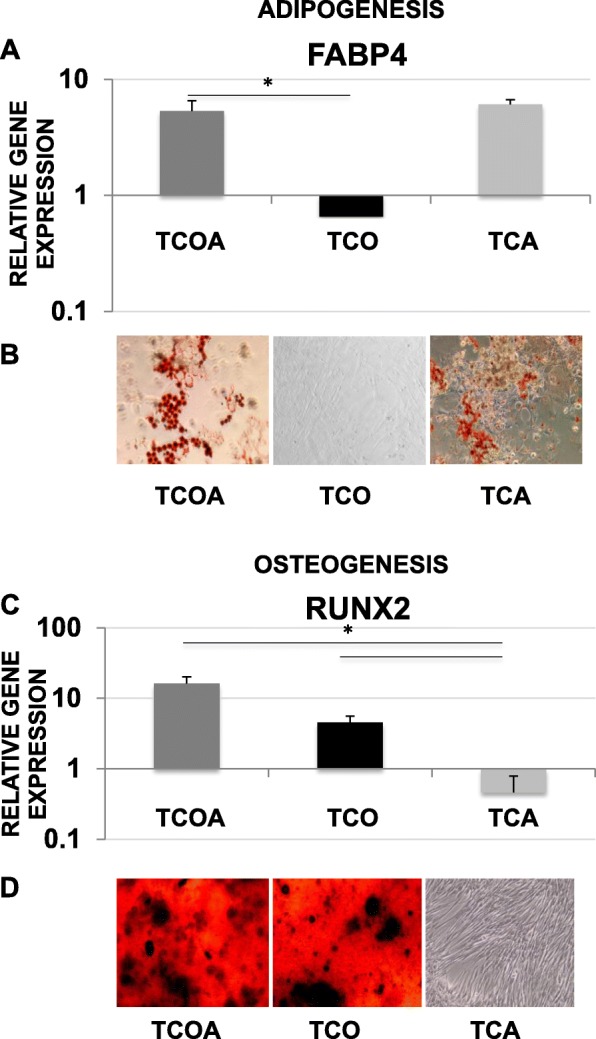
Adipogenesis assay (**a**, **b**) and osteogenesis assay (**c**, **d**) results. Gene expression of FABP4 relative to undifferentiated monolayer cells on day 21 (**a**), visualization of oily droplets in representative TCOA and TCA cultures by Oil Red O staining (**b**), gene expression of Runx2 relative to undifferentiated monolayer cells on day 21 (**c**), and visualization of calcium nodules and calcified matrices in representative TCOA and TCO cultures by Alizarin Red S staining (**d**). Lines and asterisk indicate groups with significant differences. Images are at a × 10 magnification. T, tenogenesis positive; C, chondrogenesis positive; O, osteogenesis positive; A, adipogenesis positive

**Fig. 4:**
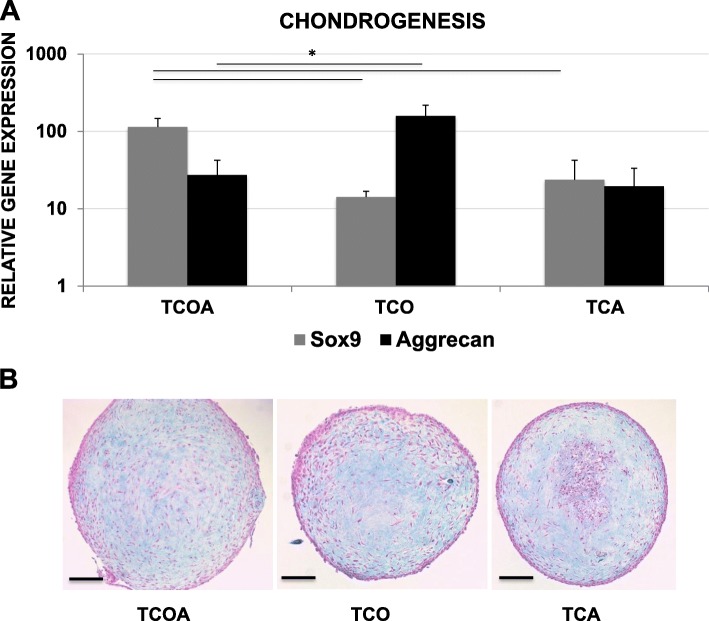
Chondrogenesis assay results. **a** Gene expression of Sox9 and aggrecan relative to uninduced monolayer cells on day 21. Lines and asterisks indicate groups with significant differences. **b** Representative sections from chondrogenesis pellets from each group showing Alcian Blue staining of the ECM. Scale bar = 500 μm

No uni-potent or bi-potent clonal TSC lines were obtained in the experiment, as each cell line differentiated toward three or four tissue types. All of the fifteen clonal TSC lines exhibited both chondrogenic and tenogenic potential, evident by the positive upregulation of tissue markers upon differentiation. Eight out of fifteen lines differentiated toward tendon, cartilage, bone, and adipose lineages (quadra-potent TCOA phenotype). Five of fifteen were positive for tenogenesis, chondrogenesis, and osteogenesis (TCO phenotype), but not adipogenesis, and did not express FABP4 (Ct ≥ 35, no amplification) (Fig. 3a). The difference in FABP4 expression between TCOA and TCO reached significance (*p* = 0.0002) and between TCA and TCO approached significance (*p* = 0.0599). Two of fifteen were positive for tenogenesis, chondrogenesis, and adipogenesis (TCA phenotype) but did not undergo osteogenesis and did not express Runx2 (Ct ≥ 35) (Fig. 3c). Runx2 expression was significantly decreased in the TCA group and compared to the TCOA (*p* = 0.0010) and TCO (*p* = 0.0179) groups. Oil Red O staining confirmed the presence of intracellular oily droplets in adipogenic cultures of the TCOA and TCA groups (Fig. 3b). Likewise, osteogenic differentiation was confirmed with the formation and staining of calcium nodules and a calcified matrix with Alizarin Red S in cultures of the TCOA and TCO groups (Fig. 3d). And, Alcian Blue staining of paraffin-embedded sections of the chondrogenesis pellets showed blue staining of proteoglycan in the ECM (Fig. 4b).

### Tissue marker gene expression levels and proliferative capacity of TSCs correlates with the TCA, TCO, and TCOA phenotypes

Gene expression of chondrogenic markers significantly differed among the three phenotypic groups (Fig. 4). Expression of chondrogenic Sox9 was significantly increased in the TCOA group compared to TCA and TCO (*p* = 0.0167 and 0.0017) on day 21 of chondrogenesis, whereas message levels of aggrecan were the highest in the TCO group and significantly greater than TCOA (*p* = 0.029). There were no significant differences in Runx2 expression between the TCOA and TCO groups (Fig. 3c) or in FABP4 expression between the TCOA and TCA groups (Fig. 3a).

**Fig. 5:**
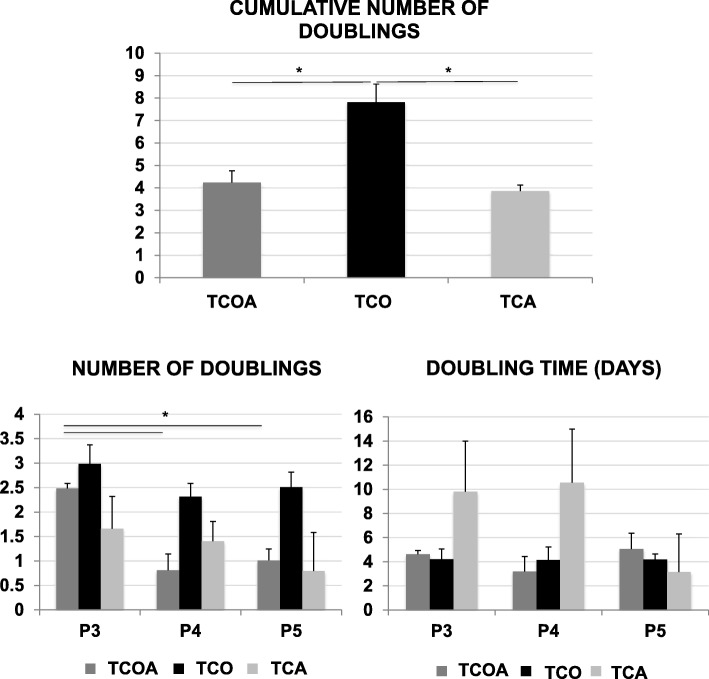
Population doubling assay results. Cumulative clonal tendon stem cell lines’ doubling numbers (top), number of doublings (bottom left), and doubling time (bottom right) over 3 successive passages. Lines and asterisks indicate groups with significant differences

The TCO group exhibited significantly greater numbers of cumulative population doublings over three passages compared to the TCOA and TCA groups (*p* = 0.0105 and 0.0392) (Fig. 5). Population doubling numbers decreased in passages 4 and 5 relative to passage 3 in all three groups, and this difference reached significance with the TCOA group.

### Quadra-differentiation potent TSCs differentiate to express the optimal composite tendon phenotype

**Fig. 6:**
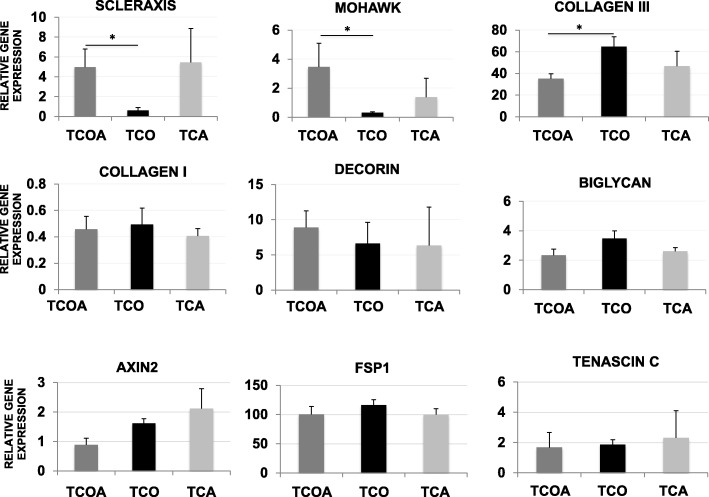
Tenogenesis assay results. Gene expression of tendon-related genes relative to equine juvenile tendon on day 10. Lines and asterisks indicate groups with significant differences

Significant differences were noted in tendon gene expression between the TCOA and TCO groups (Fig. 6). Specifically, scleraxis and mohawk were expressed > 3-fold higher in the TCOA group compared to the TCO group (*p* = 0.0454 for scleraxis and 0.0431 for mohawk). Expression of collagen type III was significantly elevated in the TCO group compared to TCOA (*p* = 0.0315). In contrast, collagen type I expression remained unaffected by TSC differentiation phenotype. No between group differences in tenascin C, Axin2, or FSP1 expression was observed.

**Fig. 7:**
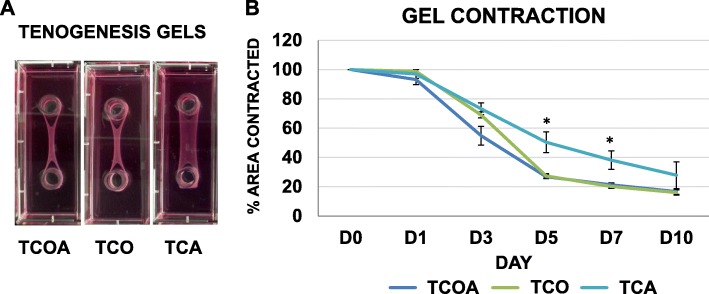
**a** Digital photographs of tenogenesis gels from each group on day 10. Gels originally filled the entire rectangular well. The tendon cells contracted the gel around the two stationary cylinders, placed 3 cm apart, to form a tendon construct. **b** Percentage contracted gel area relative to day 0 gel area at different time points over 10 days of culture. The TCA group had contracted the least at culture endpoint. Asterisks indicate groups with significant between-group differences in contraction at time point

All groups contracted collagen matrix in three dimensions albeit to different degrees over 10 days (Fig. 7). Significant differences were noted in contracted gel area at two time points between groups (Fig. 7a). Specifically, the TCA group was significantly less contracted than the TCOA and TCO groups on day 5 (*p* = 0.0004 and 0.0008) and day 7 (*p* = 0.0031 and 0.0026).

**Fig. 8:**
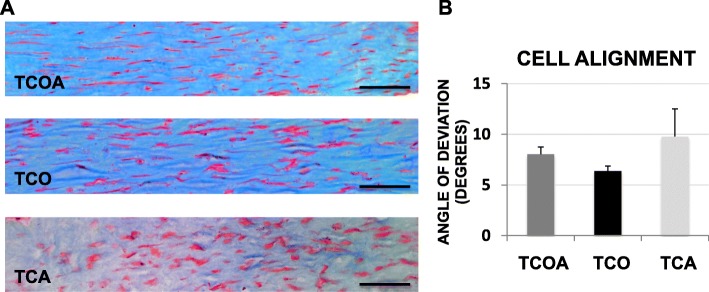
Masson’s trichrome staining of 6-μm-thick histological sections of representative tenogenesis constructs (**a**) and average cellular angle of deviation from the longitudinal axis (**b**). For cell alignment, an angle of 0° demarcates perfect parallel alignment. Scale bar on images represents 250 μm

All groups exhibited a uniform distribution of TSCs in three dimensions on day 10 (Fig. 8). A greater proportion of TSCs (> 90%) in all groups were highly aligned to the axis of tension (Fig. 8a) and exhibited elongated cell morphologies. No significant differences in cell alignment were observed between the three groups (Fig. 8b).

## Discussion

The goal of this study was to determine whether clonal TSC lines obtained from individual TSCs are diverse in their phenotypic character and the functional contributions of each phenotype to in vitro differentiation and proliferation. Three distinct TSC phenotypes were identified, and the most significant differences correlated to the presence or lack of adipogenic potential. TSCs of the TCOA phenotype strongly differentiated to a composite tendon-like construct, most remarkably by a significant increase in their expression of scleraxis and mohawk. In contrast, TSCs of the TCO phenotype exhibited significantly increased proliferative capacity compared to the TCOA and TCA groups.

This is the first study to investigate the tenogenic potentials of individual TSCs or multipotent clonal TSC lines from any species. In the absence of tendon-specific markers, we evaluated a range of markers that collectively identify the tendon phenotype [12, 24]. Significant increases in message levels of the bHLH transcription factor scleraxis [25] in the TCOA group can be attributed to tendon neogenesis [26]. Further, scleraxis and mohawk (a member of the TALE superclass of homeobox genes [27]) are co-expressed in mature, differentiated tendons [28, 29], which may explain their overlapping expression patterns observed in this study. Collagen type I is the predominant tendon collagen [30], and the ratio of collagen type III protein to type I protein is a determinant of the pathological state of tendons [31]. The higher collagen type III to type I ratio message level observed in the TCO group (compared to TCOA) may be suggestive of disorganized collagenous matrices undergoing active remodeling [31, 32], as opposed to relatively mature tendons of the TCOA group. The small leucine-rich proteoglycans decorin and biglycan are functionally similar and likely compensate for each other in vivo [33]. However, in this study, we did not achieve significant differences in their expression patterns.

A recent study revealed the presence of a highly proliferative subpopulation of Axin2 and scleraxis co-expressing TSCs at the site of healing in injured tendons, suggesting this subpopulation of cells may be the first responders to tendon injury in vivo [34]. Our results suggest that TSCs of the highly proliferative, Axin2-expressing TCO phenotype may represent this subpopulation. In contrast, scleraxis expression was significantly greater in the TCOA compared to the TCO group. This result is in support of the hypothesis that Wnt signaling suppresses tenogenic differentiation in TSCs, specifically by downregulating the expression of scleraxis [35].

When suspended in collagen hydrogel around 2 fixed points, TSCs contract a disorganized collagenous matrix to an anisotropic, tendon-like structure with parallel-aligned cells and collagen fibers [36–39]. Our tenogenesis assay successfully and consistently generated tendon-like constructs with aligned cells of elongated morphologies on day 10 of culture. The reduced contraction of the TCA group in this study suggests that downregulation of Runx2 expression in TSCs may impede tendon repair in vivo*.* One study suggests that overexpression of Runx2 may augment tendon-to-bone healing, by inducing site-specific rather than heterotopic bone formation [40].

Expansion of single-cell derived colonies is known to be difficult in vitro and limited this study to fifteen clonal TSC lines. Feeder layers, growth factors, or alternative methods to supplement cell growth were not used in this study to avoid potential interference with study outcomes. However, sufficient cell numbers were acquired from fifteen TSCs to enable gene expression analysis as a superior quantitative measure of differentiation as opposed to stain absorbance. In contrast to a previous study on tri-lineage differentiation of TSCs [9], we did not identify clones that were solely uni-potent (adipogenic, osteogenic, or chondrogenic). This may be attributed to species-related differences or the requirement of highly proliferative clones to simultaneously assess tri-lineage, tenogenic, and proliferative capacities.

Interestingly, the group with the highest differentiation capacity, TCOA, did not have the highest proliferation capacity in our study. The TCOA group exhibited significantly lower numbers of cumulative cell doublings compared to the TCO group. This observation is in contrast to the proposed model of hierarchical loss of potential in bone marrow MSCs, wherein multi-differentiation and proliferative potential are positively correlated [41, 42]. In this model, adipogenic potential is reduced with successive population doublings [43]. In the tendon, we propose that lack of adipogenic potential may signify (1) increase in proliferative potential and (2) decrease of tendon-related gene expression upon tenogenesis as observed in TSCs of the TCO phenotype. Further, it is also possible that in contrast to proposed models of lineage commitment, distinct TSC phenotypes identified in this study represent independent tendon cell populations rather than ones derived from a common ancestor. Future investigation is warranted to further understand the relationships in TSC characteristics and the molecular mechanisms underlying loss or gain of potential in vitro.

## Conclusions

Of the three TSC phenotypes identified in this study, TSCs of the TCOA phenotype had optimal tenogenic capacity, evident by the strongest expression of scleraxis and mohawk, progressive contraction over 10 days, and elongated cell morphologies in histologic sections of tendon constructs. In contrast, TSCs of the TCO phenotype may exhibit reduced osteogenesis compared with TCOA and thus offer select advantages in tendon healing such as a reduced proclivity to ectopic bone formation. Future studies may be targeted to the identification of additional TSC phenotypes and the contributions of each phenotype to functional tendon repair in vivo.

## Supplementary information

**Additional file 1.** Fig. 1 Gene expression of the transcription factor Oct-4 in clonal TSC lines.

## Data Availability

All data generated and analyzed during this study are included with this submitted article (and its supplementary information files).

## Abbreviations

TSC: Tendon stem cell
MSC: Mesenchymal stem cell
FABP4: Fatty acid binding protein-4
FSP-1: Fibroblast specific protein-1
DN: Doubling number
DT: Doubling time
CDN: Cumulative doubling number
3D: Three-dimensional
Oct-4: Octamer-4
