# Supplementary figures and images for: Multi-differentiation potential is necessary for optimal tenogenesis of tendon stem cells
Source: Stem Cell Res Ther. 2020 Apr 9;11:152. doi: 10.1186/s13287-020-01640-8 (PMC7146987; doi:10.1186/s13287-020-01640-8)

## Slide 1
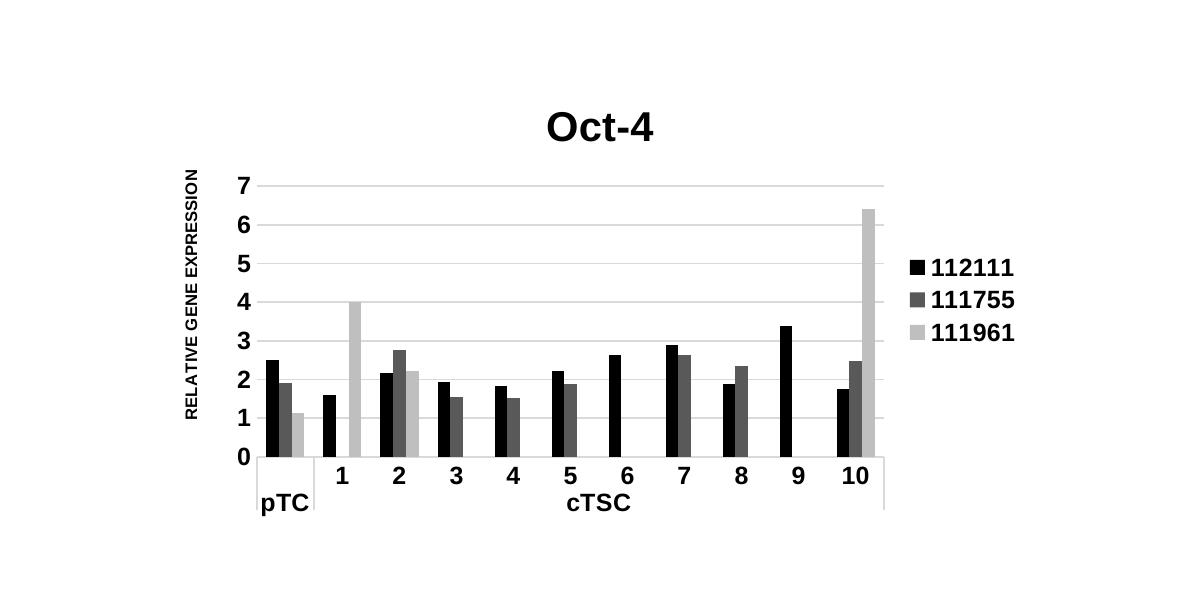

### Chart: Oct-4
| Category | 112111 | 111755 | 111961 |
|---|---|---|---|
| | 2.5 | 1.9 | 1.12 |
| 1 | 1.6021397551792478 | None | 4.0 |
| 2 | 2.1734697250521178 | 2.7702189362218537 | 2.23 |
| 3 | 1.93187265784969 | 1.5368751812880095 | None |
| 4 | 1.8276629004588023 | 1.5157165665103958 | None |
| 5 | 2.2191389441356932 | 1.892115293451194 | None |
| 6 | 2.6207868077167404 | None | None |
| 7 | 2.8878583910450013 | 2.6390158215457924 | None |
| 8 | 1.8921152934511987 | 2.3456698984637523 | None |
| 9 | 3.386981249450129 | None | None |
| 10 | 1.74 | 2.4794153998779707 | 6.4 |

Supplement: Supplementary file 1 — Additional file 1. Fig. 1 Gene expression of the transcription factor Oct-4 in clonal TSC lines. [file 13287_2020_1640_MOESM1_ESM.pptx]
